# A systematic review of staging systems for oral submucous fibrosis: evolution, variations, and clinical implications

**DOI:** 10.3389/froh.2026.1821211

**Published:** 2026-04-29

**Authors:** Prathima Shetty, M. Subhikshaa, K. M. Veena, Prashanth Shenoy, K. Sundeep Hegde, Rachana Prabhu

**Affiliations:** Yenepoya Dental College, Yenepoya University, Mangalore, India

**Keywords:** clinical, histopathological, mouth opening, oral submucous fibrosis, OSMF, staging systems

## Abstract

**Introduction:**

Multiple clinical, functional, and histopathological staging frameworks for OSMF have been described in the literature. Staging helps clinicians assess prognosis, tailor interventions, and monitor progression. The aim of the study is to collect and analyze the various staging systems for OSMF reported in the literature to date and to identify how these systems have evolved over time.

**Materials and methods:**

A comprehensive search using multiple electronic databases and gray literature was undertaken to synthesize evidence on existing staging systems. Two reviewers independently performed the study search and selection process.

**Results:**

Eighteen articles (18 staging systems) were finally included. Most frameworks used criteria such as clinical functional (mouth opening) and histopathological features (collagen deposition, fibroblast response, and inflammation) for staging, either individually or in combination. We observed that articles primarily focused on the clinical and histopathological features (1950–1970), functional limitations such as trismus and cheek flexibility (1980–1990), and integrated systems (after 2000).

**Discussion:**

Despite many available staging methods, no single system has yet gained universal acceptance due to heterogeneity in criteria, lack of validation, and variability in clinical-pathological correlations. The review underscores the critical need for standardised, evidence-based staging systems that combine clinical functionality with pathological assessment to improve diagnostic precision, prognostic evaluation, and treatment planning.

**Systematic Review Registration:**

https://www.crd.york.ac.uk/prospero/display_record.php?ID=CRD420251134270, PROSPERO CRD420251134270.

## 1 Introduction

Oral submucous fibrosis (OSMF), which is a potentially malignant disorder, mentioned in “Shushrutha” (ancient medical text) as “vidari”, which is classified under the mouth and throat diseases, (1). Schwartz in 1952 reported the first case as “Atropica idiopathica mucosae oris”. Joshi introduced the terminology “Oral Submucous Fibrosis” (OSMF) in 1953. Jens J. Pindborg defined OSMF as “an insidious, chronic disease that affects any part of the oral cavity and sometimes the pharynx. Although occasionally preceded by, or associated with, the formation of vesicles, it is always associated with a juxta-epithelial inflammatory reaction followed by fibroelastic change of the lamina propria and epithelial atrophy that leads to stiffness of the oral mucosa and causes trismus and an inability to eat.” in 1966 (2).

Recently, OSMF has been globally accepted as an Indian disease, which is due to the high prevalence in the Indian subcontinent (3). The pooled global prevalence of OSMF is 4.47%, and with higher prevalence of 6.36% in India. The primary risk factor for OSMF is chewing areca nut, and other risk factors include deficiencies in vitamins B, C, and iron, consumption of spicy foods, human papillomavirus (HPV) infection, and genetic mutations (4).

Arecoline which is present in betel nuts along with copper triggers inflammation, impairs fibroblast function, and promotes excessive deposition of collagen, resulting in fibrosis progressed to trismus. Clinically this condition presents with symptoms like burning sensation, vesicle formation, restricted oral movements, depapillation of the tongue, blanching with leathery texture of the oral mucosa, resulting in mouth opening reduction, and a shrunken uvula (5, 6).

Literature reveals that OSMF is associated with significant morbidity and a notable risk of malignant transformation rate in OSMF; leading to ongoing efforts to develop effective management (6). The management of OSMF largely determined by the of the disease. Early stages can be managed with nutritional and medical approaches, whereas moderately advanced stages require surgical management (7, 8).

The literature indicates that Desa JV (1957) was the first to propose a staging system for OSMF, after which several authors have proposed different staging systems for OSMF based on varying parameters (9).

Despite these efforts, there is still no reliable staging system. Understanding each individual staging system and its evolutionary pattern is essential for identifying challenges in applying them in practice for OSMF treatment planning. This research addresses these gaps by aggregating and critically reviewing various staging systems for OSMF reported in the literature to date and identifying the evolutionary pattern of these systems over time.

## 2 Material and methods

### 2.1 Protocol

This systematic review was conducted in accordance with the Preferred Reporting Items for Systematic Reviews and Meta-Analyses (PRISMA) statement for reporting systematic reviews. The systematic review protocol was registered on PROSPERO with the **PROSPERO registration ID: CRD420251134270.**

### 2.2 Focused question

**“**What are the different staging systems of OSMF that are listed in the literature to date, and how have they evolved?”.

The research question for the present systematic review was formulated in accordance with the PICO criteria (Table 1).

**Table 1:** Pico criteria for the current systematic review.

P	Population	Patients with oral submucous fibrosis
I	Intervention	Different Staging Systems
C	Comparator	Nil
O	Outcomes	Compile and identify the evolutionary patterns of different OSMF staging systems.

### 2.3 Information sources

The literature search was primarily conducted through electronic databases, including PubMed/Medline, Google Scholar, Scopus, Embase, EBSCO, the Cochrane Oral Health Group’s Trials Register, the Cochrane Central Register of Controlled Trials (CENTRAL), PROSPERO, and Web of Science. The search was restricted to studies articles published up to August 31, 2024, to ensure inclusion of relevant literature pertaining to OSMF staging systems. Only articles published in English, or with a detailed summary in English were considered. In addition, a manual search of relevant oral pathology, maxillofacial, dental, and medical journals was performed. Reference lists of both included and excluded studies were also screened to identify any additional relevant publications.

A combination of keywords—[“Oral Submucous Fibrosis” (Title/Abstract) OR “OSMF” (Title/Abstract) OR “Atropica idiopathica mucosae oris” (Title/Abstract)] AND [“Staging system” (Title/Abstract) OR “grading” (Title/Abstract) OR “grading system” (Title/Abstract) OR “staging” (Title/Abstract) OR “clinical” (Title/Abstract) OR “histopathological” (Title/Abstract) OR “functional” (Title/Abstract)] was applied across all databases to ensure comprehensive identification and screening of relevant articles.

### 2.4 Eligibility criteria

Studies reporting various staging systems of OSMF published to date were included. This comprised of case studies, epidemiological studies, cross-sectional studies, and clinicopathological or histopathological studies on OSMF in which authors proposed specific staging systems. Only articles published in English were included.

Systematic reviews [with or without meta-analysis], case reports, case series, expert opinions, non-randomised controlled trials, case-control, and cohort studies were excluded. Animal and *in vitro* studies were excluded. Articles with incomplete data and studies presenting duplicate or previously reported classifications were excluded. Additionally, abstracts, editorials, letters, correspondences, web content, media files, and book chapters were excluded, as were articles published in languages other than English.

### 2.5 Study selection and data extraction

The study selection process was carried out in two-stages. In the initial phase, two reviewers independently screened the titles and abstracts of the identified articles. Duplicate records were removed using Rayyan software. Based on predefined inclusion and exclusion criteria, the studies were categorized as relevant or irrelevant, and those not meeting the eligibility criteria were excluded.

In the second stage, the full text of the selected studies was assessed and re-evaluated according to the same criteria. Any discrepancies between reviewers were identified and resolved through discussion with the involvement of a third author when required.

From the final set of the included studies, key information was extracted, including the characteristics (authors and the year of publication) and details of the proposed staging systems. Data extraction was performed independently by two reviewers, tabulated manually and recorded in MS excel sheet. The extracted data was subsequently cross-verified by another reviewer, and summarized.

### 2.6 Risk of bias and quality assessment

The methodological quality of the included studies was evaluated using the Critical Appraisal Skills Programme (CASP) Checklist for clinical prediction rule (CPR) (Table 4). The tool comprises 11 questions organized into three sections and allows responses to be recorded as “Yes”, “No”, or “Unclear” (22).

## 3 Results

The inter-reviewer agreement was high, with a kappa value of 0.88, indicating strong concordance among the three investigators. A total of 9,543 articles were identified through electronic database searches, along with an additional 149 records from other sources. Following the removal of duplicates, 2,065 articles remained for screening.

**Figure 1:**
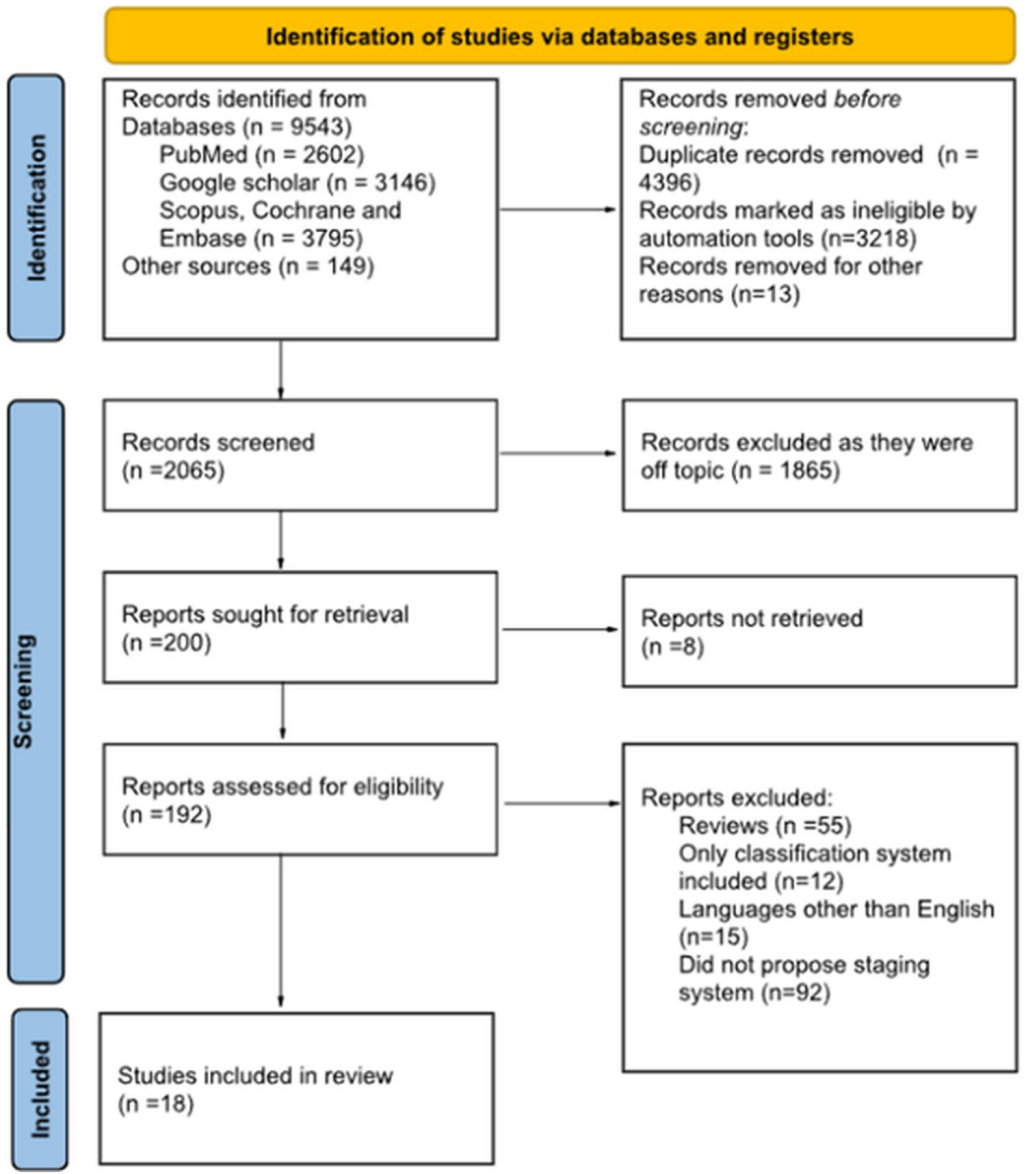
Flowchart representing the process of study selection.

After applying the eligibility criteria, the number of studies was reduced to 18, which were ultimately included in the systematic review. The study selection process is illustrated in the PRISMA flowchart (Figure 1).

### 3.1 Characteristics of included studies

A total of eighteen staging systems were included in the review. (Tables 2, 3) (2.9-25) These staging systems range from schemes based solely on mouth opening (interincisal distance) to multiparameter systems that aim to stratify disease severity and guide treatment. Functional assessment, especially mouth opening, remains the key parameter, as it is a practical and reliable marker utilized in the majority of systems. In addition to mouth opening, certain functional staging systems also assess the tongue protrusion and cheek flexibility are to determine the degree of oral restriction. Clinically staging system further relies on the presence, distribution, and location of fibrous bands in the oral mucosa particularly in the buccal, faucial, and labial regions. Moreover, histopathological parameters, including collagen deposition, epithelial atrophy, and inflammation changes have been integrated into several staging systems.

**Table 2:** Staging systems of the OSMF in the included articles.

S.no	Author name and year	Staging system	Clinical Relevance and the criteria used for staging
1	More et al., (9)	“Stage I: Stomatitis and vesiculation.	Utilized features such as the presence of vesicles, stomatitis, fibrotic bands, and any premalignant and malignant changes.
		Stage II: Fibrosis.	
		Stage III: Sequelae of the disease.”	
		More et al, (9)	
			No Histological or functional features such as mouth opening correlation
2	Pindborg JJ and Sirsat (2)	**“Very early stage**: Finely fibrillar collagen dispersed with marked oedema. Plump young fibroblast containing abundant cytoplasm. Blood vessels are dilated and congested. Inflammatory cells, mainly polymorphonuclear leukocytes with occasional eosinophils, are found. **Early stage:** the juxta-epithelial area shows early hyalinisation. Collagen is still in separate thick bundles. A moderate number of plump young fibroblasts is present. Dilated and congested blood vessels. Inflammatory cells are primarily lymphocytes, eosinophils, and occasional plasma cells. **Moderately advanced stage**: Collagen is moderately hyalinised. Thickened collagen bundles are separated by a slight residual oedema. Fibroblastic response is less marked. Blood vessels are either normal or compressed. Inflammatory exudate consists of lymphocytes and plasma cells. **Advanced stage**: Collagen is completely hyalinised. Smooth sheets without separate collagen bundles are seen. Oedema is absent. The hyalinised area is devoid of fibroblasts. Blood vessels are completely obliterated or narrowed. Inflammatory cells are lymphocytes and plasma cells.”	Depending on collagen hyalinisation, the inflammatory cell infiltrate
		Pindborg et al., (2)	
3	Gupta et al. (11)	**“Very early stage**: Complaints of burning sensation in the mouth or ulceration without any difficulty opening the mouth **Early stage:** Along with a burning sensation, complaints of slight difficulty opening the mouth. **Moderately advanced stage:** Marked trismus, to the extent that the patient cannot open their mouth more than two finger-widths. Associated difficulties with mastication are apparent. **Advanced stage:** Patient is undernourished and anaemic, and shows marked trismus and/or other symptoms, as mentioned above”	According to the increasing intensity of trismus
		Gupta et al. (11)	
4	Pindborg JJ. (12)	“Stage I: Stomatitis includes erythematous mucosa, vesicles, mucosal ulcers, melanotic mucosal pigmentation, and mucosal petechiae.	Depending on the stomatitis-involved sites in the oral cavity, fibrosis, pigmentation, and petechiae.
		Stage II: Fibrosis occurs in healing vesicles and ulcers, which is the hallmark of this stage.	
		Early lesions show blanching of the oral mucosa. Older lesions include vertical and circular palpable fibrous bands in the buccal mucosa and around the mouth opening or lips. This results in a mottled marble-like appearance of the mucosa because of the vertical, thick, fibrous bands in association with a blanched mucosa. Specific findings include reduction of mouth opening, stiff and small tongue, blanched and leathery floor of the mouth, fibrotic and depigmented gingiva, rubbery soft palate with decreased mobility, blanched and atrophic tonsils, shrunken bud-like uvula, and sunken cheeks, not commensurate with age or nutritional status.	
		Stage III: Sequelae of OSMF are as follows:	
		Leukoplakia is found in more than 25% of individuals with OSMF. Speech and hearing deficits may occur because of the involvement of the tongue and the eustachian tube.” Pindborg et al., (12)	
5	Nagesh and Bailoor (13)	“Stage I early OSMF:	Depending on the diagnosis
		Mild blanching, no restriction in mouth opening (normal distance between central incisor tips: Males 35 to 45 mm, females 30 to 42 mm), no restriction in tongue protrusion (normal mesioincisal angle of upper central incisor to the tip of the tongue when maximally extended with the mouth wide open): males 5 to 6 cm, females 4.5 to 5.5 cm. Cheek flexibility CF = V1–V2, two points measured between; V2 is marked at 1/3rd the distance from the angle of the mouth on a line joining the tragus of the ear and the angle of the mouth, and V1 is the subject. The subject is then asked to blow his cheeks fully, and the distance is measured between the two points marked on the cheek. Mean value for males = 1.2 cm; females = 1.08 cm. Burning sensation on taking spicy food or hot beverages.	
		Stage II moderate OSMF:	
		Moderate to severe blanching, Mouth opening reduced by 33%, Cheek flexibility was also demonstrably reduced. Burning sensation also in the absence of stimuli, Palpable bands felt. Lymphadenopathy, either unilateral or bilateral, and demonstrable anaemia on haematological examination.	
		Stage III severe OSMF:	
		The burning sensation is very severe, and the patient is unable to do day-to-day work. more than 66% reduction in the mouth opening, cheek flexibility, and tongue protrusion. The tongue may appear fixed. Ulcerative lesions may appear on the cheek Thick palpable bands are bilaterally evident Lymphadenopathy is bilaterally evident.”	
		Nagesh and Bailoor et al., (13)	
6	Mathur and Jha (14)	“Stage 1: Early OSF	Clinical features of OSF
		Mild blanching No restriction in mouth opening. No restriction in tongue protrusion, measuring from the mesio-incisal angle of an upper central incisor to the tip of the tongue when maximally extended with the mouth at maximal opening. Burning sensation only on ingesting spicy foods, hot liquids, etc.	
		Stage 2: Moderate OSF	
		Moderate to severe blanching Mouth opening was reduced by 33%, tongue protrusion was reduced by 33%, and flexibility also demonstrably decreased. Burning sensation even in the absence of stimuli. Presence of palpable bands. Lymphadenopathy, either unilateral or bilateral. Demonstrable anaemia on haematological examination.	
		Stage 3: Severe OSF	
		Very severe burning sensation; the patient is unable to perform day-to-day work. More than 66% reduction in mouth opening, cheek flexibility, and tongue protrusion. In many cases, the tongue may appear fixed. Ulcerative lesions may appear in the cheek. Thick palpable bands. Lymphadenopathy is evident bilaterally.”	
		Mathur and Jha et al., (14)	
7	Mehata and Hammer (36)	“Stage 1: recurrence, burning sensation, ulceration, and amount of fibrosis.	Categorized depending on habits
		Stage 2: Mouth opening, tongue protrusion, fibrosis, colour of lips and cheeks,	
		Stage 3: Leukoplakia, ulceration, and malignant lesions may be seen on involved sites; precancerous condition; atrophy of epithelium; epithelium undergoes more malignant changes.”	
		Mehata and Hammer (36)	
8	Khanna and Andrade (15)	“Stage 1	Divided into clinically and histologically
		Interincisal opening of 35 mm and above Burning sensation in the mouth Acute ulceration and recurrent stomatitis	
		Stage 2	
		Interincisal opening of 26–35 mm Mottled and marble-like buccal mucosa Dense, pale, depigmented, fibrosed areas alternated with pink, normal mucosa Occasional red erythroplakia patches Widespread sheets of fibrosis	
		Stage 3	
		Interincisal opening of 15–25 mm Pale buccal mucosa firmly attached to the underlying tissues Palpable vertical fibrous bands in the premolar area Unable to blow out cheeks and whistle In the soft palate, the fibrous bands were seen to radiate from the pterygomandibular raphe or the anterior faucial pillar in a scar-like appearance The lips may be affected with atrophy of the vermilion border	
		Stage 4 (4a and 4b)	
		Interincisal opening of 15 mm or less Thickened, shortened, and firm fauces, with the tonsils compressed between the fibrosed pillars Small, shrunken, fibrous bud uvula Narrowed isthmus, presence of a circular band around the entire lip and mouth Restricted tongue movement, diffuse papillary atrophy Atrophy of the vermilion border Premalignant and malignant changes”	
		Khanna and Andrade et al., (15)	
9	Haider et al. (16)	“Clinical Staging:	Clinically and functionally.
		Stage 1: faucial bands only	
		Stage 2: faucial and buccal bands	
		Stage 3: faucial and labial bands	
		Functional Staging:	
		Stage A: mouth opening, 13–20 mm	
		Stage B: mouth opening, 10–12 mm	
		Stage C: mouth opening < 10 mm”	
		Haider et al. (16)	
10	Utsunomiya et al. (23)	“Early stage: a large number of lymphocytes in the subepithelial connective tissue zone, along with myxedematous changes.	Histopathological
		Intermediate stage: Granulation changes close to the muscle layer, and hyalinization appears in the subepithelial zone, where blood vessels are compressed by fibrous bundles. Reduced inflammatory cells in the subepithelial layer.	
		Advanced stage: Inflammatory cells are hardly seen. The number of blood vessels is dramatically small in the subepithelial zone. Marked fibrous areas with hyaline changes extending from subepithelial to superficial muscle layers. Atrophic, degenerative changes start in muscle fibers.”	
		Utsunomiya et al., (23)	
11	Kiran Kumar et al. (17)	“Stage I: Mouth opening >45 mm	Clinical stages of OSMF
		Stage II: Restricted mouth opening 20 to 44 mm	
		Stage III: Mouth opening < 20 mm”	
		Kiran Kumar et al., (17)	
12	Chandramani More et al., (10)	“Clinical staging:	Clinical and functional
		Stage 1 (S1): Stomatitis and/or blanching of the oral mucosa. Stage 2 (S2): Presence of palpable fibrous bands in buccal mucosa and/or oropharynx, with/without stomatitis. Stage 3 (S3): Presence of palpable fibrous bands in buccal mucosa and/or oropharynx, and in any other parts of the oral cavity, with/without stomatitis. Stage 4 (S4) is as follows: a. Any one of the above stages along with other potentially malignant disorders, e.g., oral leukoplakia, oral erythroplakia, etc. b. Any one of the above stages, along with oral carcinoma.	
		Functional staging:	
		M1: Interincisal mouth opening up to or greater than 35 mm. M2: Interincisal mouth opening between 25 and 35 mm. M3: Interincisal mouth opening between 15 and 25 mm. M4: Interincisal mouth opening less than 15 mm.	
		Example—S1M1, S2M3, S2M4, S3M4, S4AM2, S4BM3.”	
		Chandramani More et al., (10)	
13	Santosh Patil and Sneha Maheshwari et al., (18)	“Grade 1 [Early]: Cheek flexibility of 30 mm and above	Cheek flexibility
		Grade 2 [Mild]: Cheek flexibility between 20 and 30 mm	
		Grade 3 [Moderate]: Cheek flexibility less than 20 mm	
		Grade 4 [Severe]: Any of the above conditions without concurrent presence of potential malignant lesions	
		Grade 5 [Advanced]: Any of the above conditions with concurrent presence of oral carcinoma”	
		Santosh Patil and Sneha Maheshwari et al., (18)	
14	Rajinikanth et al., (37)	“Stage I:	Clinical, functional, and histopathological
		Clinically, patients complain of a burning sensation and altered taste perception. The opening of the mouth can be scored as 36 mm to 40 mm. When related to buccal mucosa, symptoms are not present. Histopathologically, Collagen fibers are finely spread with noticeable edema, with a huge number of plump young fibroblasts along with abundant cytoplasm. In the connective tissue stroma, inflammatory cells are composed mainly of lymphocytes and eosinophils. Rarely do we see eosinophils. The overlying epithelium is normal.	
		Stage II:	
		Clinically: The patient complains of a burning sensation, increased sensitivity to spicy food, and white band-like lesions that can be seen on any one anatomical site in the oral cavity. Mouth opening can be scored as 32 mm to 36 mm. Histopathologically, collagen is still separate and thick with separate bundles. Juxta-epithelial hyalinisation is present. It contains young fibroblasts in moderate count. They contain dilated blood vessels. Inflammatory cells consist of lymphocytes and a few eosinophils. They rarely contain plasma cells. The epithelium shows flattening or shortened epithelial rete pegs. They are evident with varying degrees of keratinisation.	
		Stage III:	
		Clinically, the patient complains of a severe burning sensation when they take hot or spicy food. Extensive fibrous white bands on the buccal mucosa can be palpated. d. There is difficulty in mastication. Mouth opening can be scored 28 mm to 32 mm. Where patients cannot open their mouth more than two fingers of their own. Histopathologically, thick collagen bundles are separated by slight oedema. Juxta-epithelial hyalinisation is present. Connective tissue stroma consists of conjugated blood vessels, mature fibroblasts, scanty cytoplasm, and spindle-shaped nuclei. Inflammatory cells are mainly neutrophils and plasma cells. Muscle fibres are thick, and collagen fibres are dense. The epithelium is atrophic with loss of rete pegs.	
		Stage IV:	
		Clinically: The patient is anaemic and malnourished due to poor nutrition and due to the inability to open the mouth. Severe trismus, with fibrous white bands extending all over the mouth over the prominent anatomical sites. Mouth opening can be scored as less than 10 mm. Histopathologically, completely hyalinised collagen is present. This collagen is in the form of smooth sheets. Oedema is not present in this stage. Fibroblasts are absent. Connective tissue stroma consists of blood vessels, which are destroyed or restricted. Inflammatory cells are lymphocytes and plasma cells. They also show mild to moderate atypia and severe degeneration of muscle fibres.”	
		Rajinikanth et al. (37)	
15	Yesha V Jani, et al., (19)	“Stage 1	Clinical and functional
		Normal mouth opening (35–45 mm) Burning sensation only on consuming spicy or hot food Blanching of the faucial pillars, soft palate, and buccal mucosa Normal tongue protrusion No palpable fibrous bands	
		Stage II	
		Mouth opening reduced by 1/3rd (25–38 mm) Burning sensation on eating normal food Blanching of the faucial pillars, soft palate, buccal mucosa, and labial mucosa Normal tongue protrusion Palpable fibrous bands in the faucial pillars, palate, and buccal mucosa	
		Stage III	
		Mouth opening reduced by two-thirds (20–30 mm) Severe burning sensation even in the absence of stimuli Blanching of the faucial pillars, soft palate, buccal mucosa, labial mucosa, and floor of the mouth Tongue protrusion reduced Palpable fibrous bands in the faucial pillars, palate, buccal mucosa, and labial mucosa Unilateral or bilateral lymphadenopathy	
		Stage IV	
		Mouth opening 15–25 mm Severe burning sensation Blanching of the faucial pillars, soft palate, buccal mucosa, labial mucosa, and floor of the mouth Restricted tongue movement Atrophy of the tongue papilla Palpable fibrous bands in the faucial pillars, palate, buccal mucosa, labial mucosa, and floor of mouth Hyposalivation Unilateral or bilateral lymphadenopathy Reduced movements of the soft palate Uvula-small, shrunken, deviated, or fibrous”	
		Yesha V Jani et al., (19)	
16	Passi et al. (24)	“Grade 1	Clinical, functional, and histopathological integrative system
		Involvement of less than one-third of the oral cavity Mild blanching, burning sensation, recurrent ulceration, and stomatitis. dryness of mouth Mouth opening up to 35 mm Stage of inflammation: Fine edematous collagen, congested blood vessels, abundant neutrophils, along with lymphocytes, with myxomatous changes in the subepithelial connective tissue layer of epithelium	
		Grade 2	
		Involvement of one-third to two-thirds of the oral cavity Blanching of the oral mucosa with a mottled and marble-like appearance, fibrotic bands palpable, and involvement of the soft palate and premolar area Mouth opening 25–35 mm Cheek flexibility reduced by 33% Stage of hyalinisation: Juxta-epithelial collagen hyalinisation with lymphocytes, eosinophils. Dilated and congested blood vessels. Less fibroblastic activity. Granulation changes in the muscle layer with reduced inflammatory cells in the subepithelial layer	
		Grade 3	
		Involvement of more than two-thirds of the oral cavity. Severe blanching; broad, thick, fibrous palpable bands at cheeks and lips; rigid mucosa; depapillated tongue; restricted tongue movement; and shrunken budlike uvula. Floor of the mouth involvement and lymphadenopathy Mouth opening 15–25 mm Cheek flexibility reduced by 66% Stage of fibrosis: complete collagen hyalinisation without fibroblasts and oedema. Obliterated blood vessels Plasma cells and lymphocytes are present Extensive fibrosis with hyalinisation from subepithelial to superficial muscle layers with atrophic, degenerative changes.	
		Grade 4	
		Leukoplakia changes, erythroplakia Ulcerating and suspicious malignant lesion Mouth opening <15 mm or nil Stages of malignant transformation: Erythroplakia changes into squamous cell carcinoma”	
		Passi et al., (24)	
17	Arakeri et al. (20)	“Stage 1: T0–2/E0–E2 F1–3 M0/1a	Three-component staging systems
		Stage 2: T3/E3 F3–4 M0/1a/1b	
		Stage 3: Any T F1–4 M2	
		Where: Trismus (T), Fibrosis (F), Malignant Transformation (M)”	
		Arakeri et al. (20)	
18	Shaul Hameed et al. (21)	“Stage I – < 30 mm mouth opening	Functional
		Stage II—II-30–45 mm mouth opening	
		Stage III: >45 mm mouth opening”	
		Shaul Hameed et al., (21)	

**Table 3:** Segregation of the staging systems based on the staging criteria.

S.No	Staging criteria	Staging system
1	Clinical criteria	Kiran Kumar et al. (17) Mathur and Jha (14) Mehata and Hammer (36) More et al. (9) Gupta et al. (11) Pindborg (12)
2	Functional	Shaul Hameed et al. (21) Santosh Patil and Sneha Maheshwari (18)
3	Histopathological	Utsunomiya H, Tilakratne WM, Oshiro K et al. (23) Pindborg JJ and Sirsat (2)
4	Clinical and Functional	Yesha V Jani et al. (19) Chandramani More et al. (10) Haider et al. (16) Nagesh and Bailoor (13)
5	Clinical and Histopathological	Khanna and Andrade (15)
6	Clinical, histopathological, and functional	Passi D et al. (24) Ranjinikanth et al., (2016)
7	Functional and malignant transformation	Arakeri et al. (20)

From the included studies, it is observed that articles published between 1950 and 1970 focused on the clinical and histopathological features, while those published between 1980 and 1990 emphasised the functional limitations, such as trismus and cheek flexibility. Staging systems proposed after the year 2000 were mainly integrated systems combining clinical, functional, and histopathological features and malignant transformation risk.

### 3.2 Risk of bias and quality assessment

The methodological quality of the included studies was assessed using the Critical Appraisal Skills Program (CASP) Checklist for clinical prediction rule (CPR), which is summarized in Table 4 (22). All studies clearly defined the CPR in terms of patients, variables, and outcomes (Q1), indicating good clarity in study design, and most studies involved an appropriate and representative population (Q2), though a few exceptions were noted. None of the studies validated the CPR in a different patient group (Q3), reported rule performance metrics or prediction estimates, and blinded predictor variables and outcomes, highlighting a major gap in external validation. Statistical methods for rule construction and validation were clearly described in only two studies, indicating low methodological transparency across most studies. Overall, while the CPRs are well-defined and appear clinically useful, the lack of external validation, methodological clarity, and performance reporting limits confidence in their generalizability and robustness.

**Table 4:** Quality assessment of included studies by critical appraisal skills program (CASP) checklist for clinical prediction rule.

Question	More et al. (9)	Pindborg JJ and Sirsat (2)	Gupta et al. (11)	Pindborg JJ (12)	Nagesh and Bailoor (13)	Mehata and Hammer (36)	Khanna and Andrade (15)	Utsunomiya et al. (23)	Kiran Kumar et al. (17)	Mathur and Jha (14)	Haider et al. (16)	More et al. (10)	Santosh Patil and, Sneha Maheshwari (18)	Rajinikanth. et al. (37)	Yesha V Jani, et al. (19)	Passi et al. (24)	Arakeri et al. (20)	Shaul Hameed et al. (21)
Q1	✓	✓	✓	✓	✓	✓	✓	✓	✓	✓	✓	✓	✓	✓	✓	✓	✓	✓
Q2	✓	✓	✓	✓	✓	✓	✓	✓	✓	✓	✓	✓	✓	×	✓	×	✓	✓
Q3	×	×	×	×	×	×	×	×	×	×	×	×	×	×	×	×	×	×
Q4	×	×	×	×	×	×	×	×	×	×	×	×	×	×	×	×	×	×
Q5	×	×	×	×	×	×	×	×	×	×	×	×	×	×	×	×	✓	✓
Q6	×	×	×	×	×	×	×	×	×	×	×	×	×	×	×	×	✓	✓
Q7	×	×	×	×	×	×	×	×	×	×	×	×	×	×	×	×	×	×
Q8	∼	∼	∼	∼	∼	∼	∼	∼	∼	∼	∼	∼	∼	∼	∼	∼	✓	✓
Q9	✓	✓	✓	✓	✓	✓	✓	✓	✓	✓	✓	✓	✓	✓	✓	✓	✓	✓
Q10	✓	✓	✓	✓	✓	✓	✓	✓	✓	✓	✓	✓	✓	✓	✓	✓	✓	✓
Q11	✓	✓	✓	✓	✓	✓	✓	✓	✓	✓	✓	✓	✓	✓	✓	✓	✓	✓

✓ - Yes; ×- No; and∼- unclear.

## 4 Discussion

Oral submucous fibrosis is a highly prevalent premalignant condition predominantly seen in people in southern Asian countries or southern Asian immigrants to other parts of the world (25–27). The ultimate goal of classifying and staging oral submucous fibrosis is to allow physicians to plan a treatment strategy, as the choice of intervention, whether non-pharmacological, pharmacological, or surgical, depends on the stage of the disease (28–30).

Multiple clinical, functional, and histopathological staging frameworks for OSF have been described in the literature. The clinical features of OSMF include mucosal blanching, burning sensation, restricted mouth opening, rigidity, and characteristic fibrous bands (5). In most classification systems, the presence and location of the fibrous bands are the criteria for clinical staging, whereas mouth opening is an objective criterion for functional staging (31).

Some staging systems range from purely mouth-opening (interincisal distance) to multiparameter systems that aim to stratify disease severity and guide treatment. Histopathologically, fibrosis of the oral mucosa in OSMF is characterized by connective tissue changes, including deposition of dense collagen fibers (32). Thus, these criteria form the basis for the definition of this crippling disease given by Pindborg in 1966.

In the present research, eighteen staging systems were included. Among these, the commonly cited systems include those by Pindborg & Sirsat (2) (histopathology-based), Haider et al. (16) (clinical and functional), Khanna and Andrade (15) (clinical and functional), and Passi et al. (24) (integrative—clinical, functional, and histopathology-based). By reviewing these staging systems, it is evident that most frameworks divide OSMF into early, intermediate, and advanced stages, or into stages I, II, III, and IV, based on criteria such as mouth opening (in mm or percentage reduction) and the presence of fibrous bands.

Functional assessment, especially mouth opening, remains the most practical and reliable clinical marker, although it may not fully reflect the histological grade or malignant potential (16, 21). In 2000, Gururaj Arakeri et al. proposed a staging system for OSMF that includes a three-component criterion: T-trismus, F-fibrosis, and M-malignant transformation. The third component, “M”, represents the combination of suspicious clinical structural changes with histopathological features.

Hameed et al. (21) demonstrated the use of individualized criteria, by incorporating the percentage reduction in mouth opening (PRMO) relative to the patient's normal maximum mouth opening (MMO), thereby improve accuracy and consistency. This system classifies OSMF into Stage I (<30% reduction), Stage II (30%–45% reduction), and Stage III (>45% reduction), in contrast to the conventional methods that rely on the fixed mouth-opening measurement without accounting for individual variability.

Integrated staging systems, such as that proposed by Passi et al. (24), combine clinical, functional, and histopathological parameters along with the treatment guidelines and prognostic implications for each stage. While this allows for a more comprehensive assessment, it requires further validation and has limited widespread adoption.

Additionally, Modak et al. (31) reported a significant correlation between the staging system proposed by Haider et al. and the histopathological findings in OSMF specimens, stained with picro-sirius red and evaluated under polarising microscopy.

Another study demonstrated that clinically advanced OSF cases, as defined by Khanna and Andrade (15), generally correspond to more severe histopathological grades characterised by extensive fibrosis, vascular constriction, and tissue changes. Syeda Arshiya Ara (33) reported that clinical and functional staging did not show a correlation with histopathological grading, however a strong and statistically significant correlation was observed between clinical and functional staging. This discordance can occur due to biopsy site selection, disease progression patterns, and measurement.

Emerging technologies such as artificial intelligence and laser-based diagnostic tools may enhance the early detection and evaluation of Oral Submucous Fibrosis. AI-driven systems can assist in analyzing clinical images, radiological data, and histopathological findings to improve diagnostic accuracy and risk assessment. In addition, non-invasive laser-based techniques, including autofluorescence imaging, can help identify early mucosal alterations that may not be visible during routine clinical examination. The integration of these advanced technologies may facilitate earlier diagnosis, better staging assessment, and improved monitoring of disease progression and malignant transformation (34, 35).

Despite many available staging methods, the lack of uniform criteria and variability in definitions (for mouth opening, fibrosis extent, and oral site involvement) exist in the diagnostic criteria and classification parameters used across these systems, same patient may be classified into different stages by different staging systems depending on the parameters used. These variations have important clinical implications, as staging often guides treatment selection, patient monitoring, and for the assessment of disease progression which can subsequently influence clinical decision-making, treatment planning, and prognostic evaluation which hinders inter-study comparisons and real-world application and understanding the strengths and limitations of each staging system is essential for clinicians to ensure accurate assessment and appropriate management of patients in clinical practice. Overall, combining clinical, functional, histopathological, and malignant transformation criteria can provide a more complete picture of OSMF severity and progression, though discrepancies among them may occur.

The key limitations of this systematic review include heterogeneity in staging criteria, sparse validation data, and conflicting evidence regarding associations between stages and outcomes such as malignant transformation or response to interventions. Furthermore, most systems are built on small regional patient cohorts, primarily from the Indian subcontinent, which limits generalizability. Being limited to English-language studies could result in language and knowledge bias and the exclusion of pertinent data. There is a need for Greater standardization of staging criteria, an international consensus and multicenter validation that account for genetic, environmental, and ethnic factors influencing OSMF. This would aid in precise diagnosis, enhanced treatment planning, and evaluation of treatment prognosis and enhance the reliability of clinical outcomes.

## 5 Conclusion

OSMF is a progressive chronic condition linked to certain habits, and it carries a significant risk of turning malignant. Managing this complex disorder often requires a combination of treatment approaches. Staging systems play an important role in helping healthcare providers predict disease outcomes, customise treatment plans, and track how the condition evolves. This study revealed that several systems have been proposed in the literature; staging systems proposed in the last two decades were mainly integrated systems that combined clinical, functional, and histopathological features with the risk of malignant transformation. Despite the numerous staging methods proposed over time, none has become universally accepted, largely due to differences in criteria, a lack of comprehensive validation, and inconsistent correlations between clinical and pathological findings.

The review underscores the critical need for standardised, evidence-based staging systems that integrate clinical and functional outcomes with pathological assessment to improve diagnostic precision, prognostic evaluation, and treatment planning. Such an approach would enhance diagnostic accuracy, improve prognostic assessments, and lead to more effective treatment strategies. Moving forward, research should emphasise collaborative multicentre studies for validation, explore the integration of biomarkers and advanced imaging techniques, and foster consensus among experts across disciplines. Developing a widely accepted staging protocol will ultimately improve patient care, enable consistent comparisons across studies, and deepen our understanding of OSMF progression and its potential for malignant transformation. In summary, this systematic review reveals the varied and complex nature of OSMF staging systems in current literature, identifies key limitations that hinder their clinical use, and calls for cohesive, comprehensive strategies to refine disease assessment and optimise outcomes for affected patients.

## Data Availability

The original contributions presented in the study are included in the article/Supplementary Material, further inquiries can be directed to the corresponding author.

## References

[1] Shih YH Wang TH Shieh TM Tseng YH. Oral submucous fibrosis: a review on etiopathogenesis, diagnosis, and therapy. Int J Mol Sci. (2019) 20(12):2940. 10.3390/ijms20122940 31208114 PMC6627879

[2] Pindborg JJ Sirsat SM. Oral submucous fibrosis. Oral surgery, oral medicine. Oral Pathol. (1966) 22(6):764–779. 10.1016/0030-4220(66)90367-7 5224185

[3] Srivastava R Jyoti B Pradhan D Siddiqui Z. Prevalence of oral submucous fibrosis in patients visiting dental OPD of a dental college in Kanpur: a demographic study. J Family Med Prim Care. (2019) 8(8):2612–2617. 10.4103/jfmpc.jfmpc_465_19 31548942 PMC6753822

[4] Sowmya S Sangavi R. Prevalence of oral submucous fibrosis with other oral potentially malignant disorders: a clinical retrospective study. Cureus. (2023) 15(11):e49642. 10.7759/cureus.49642 38161840 PMC10755630

[5] Rao NR Villa A More CB Jayasinghe RD Kerr AR Johnson NW. Oral submucous fibrosis: a contemporary narrative review with a proposed inter-professional approach for an early diagnosis and clinical management. J Otolaryngol Head Neck Surg. (2020) 49(1):3. 10.1186/s40463-020-0399-7 31915073 PMC6951010

[6] Chhabra AK Sune R Reche A. Oral submucous fibrosis: a review of the current concepts in management. Cureus. (2023) 15(10):e47259. 10.7759/cureus.47259 38022118 PMC10655494

[7] More CB Gavli N Chen Y Rao NR. A novel clinical protocol for therapeutic intervention in oral submucous fibrosis: an evidence based approach. J Oral Maxillofac Pathol. (2018) 22(3):382–391. 10.4103/jomfp.JOMFP_223_18 30651684 PMC6306594

[8] Koneru A Hunasgi S Hallikeri K Surekha R Nellithady GS Vanishree M. A systematic review of various treatment modalities for oral submucous fibrosis. J Adv Clin Res Insights. (2014) 1(2):64–72. 10.15713/ins.jcri.18

[9] More CB Gupta S Joshi J Varma SN. Classification system for oral submucous fibrosis. J Indian Acad Oral Med Radiol. (2012b) 24(1):24. 10.5005/jp-journals-10011-1254

[10] More CB Das S Patel H Adalja C Kamatchi V Venkatesh R. Proposed clinical classification for oral submucous fibrosis. Oral Oncol. (2012a) 48(3):200–202. 10.1016/j.oraloncology.2011.10.011 22070918

[11] Gupta DS Gupta MK Golhar BL. Oral submucous fibrosis-clinical study and management by physiofibrolysis (MWD). J Indian Dent Assoc. (1980) 52(1):375–378.

[12] Pindborg JJ. Oral submucous fibrosis: a review. Ann Acad Med Singap. (1989) 18(5):603–607. 2694917

[13] Bailoor DN. Oral submucous fibrosis: the Mangalore study. IAOMR. (1993) 4:12–5.

[14] Mathur RM Jha T. Normal oral flexibility-A guideline for SMF cases. J Indian Dent Assoc. (1993) 64:139–43.

[15] Khanna JN Andrade NN. Oral submucous fibrosis: a new concept in surgical management: report of 100 cases. Int J Oral Maxillofac Surg. (1995) 24(6):433–439. 10.1016/S0901-5027(05)80473-4 8636640

[16] Haider SM Merchant AT Fikree FF Rahbar MH. Clinical and functional staging of oral submucous fibrosis. Br J Oral Maxillofac Surg. (2000) 38(1):12–15. 10.1054/bjom.1999.0062 10783440

[17] Kiran Kumar K Saraswathi TR Ranganathan K Devi MU Elizabeth J. Oral submucous fibrosis: a clinico-histopathological study in Chennai. Indian J Dent Res. (2007) 18(3):106–111. 10.4103/0970-9290.33785 17687172

[18] Patil S Maheshwari S. Proposed new grading of oral submucous fibrosis based on cheek flexibility. J Clin Exp Dent. (2014) 6(3):e255. 10.4317/jced.51378 25136426 PMC4134854

[19] Jani YV Dudhia BB. The clinicohistopathologic study of oral submucous fibrosis: a new staging system with treatment strategies. J Indian Acad Oral Med Radiol. (2016) 28(2):111–118. 10.4103/0972-1363.195082

[20] Arakeri G Thomas D Aljabab AS Hunasgi S Rai KK Hale B Tfm classification and staging of oral submucous fibrosis: a new proposal. J Oral Pathol Med. (2018) 47(4):403–409. 10.1111/jop.12689 29405430

[21] Hameed S Chatra L Shenai P. Establishing a new staging system for oral submucous fibrosis and correlation of the proposed staging system to traditional histopathological grading: a clinico-histopathological study. Saudi Dent J. (2019) 31(4):445–450. 10.1016/j.sdentj.2019.04.003 31695294 PMC6823736

[22] Critical Appraisal Skills Programme. CASP Clinical Prediction Rule Checklist (2024). Available online at: casp-uk.net. Available online at: https://casp-uk.net/casp-tools-checklists/clinical-prediction-rule-checklist/ (Accessed October 16, 2025).

[23] Utsunomiya H Tilakaratne WM Oshiro K Maruyama S Suzuki M Ida-Yonemochi H Extracellular matrix remodeling in oral submucous fibrosis: its stage-specific modes revealed by immunohistochemistry and *in situ* hybridization. J Oral Pathol Med. (2005) 34(8):498–507. 10.1111/j.1600-0714.2005.00339.x 16091118

[24] Passi D Bhanot P Kacker D Chahal D Atri M Panwar Y. Oral submucous fibrosis: newer proposed classification with critical updates in pathogenesis and management strategies. Natl J Maxillofac Surg. (2017) 8(2):89–94. 10.4103/njms.NJMS_32_17 29386809 PMC5773997

[25] Evanjelin PJ Maheswari TU Salam S Tripathi S Pandey A Bahadure P Prevalence of different stages of oral submucous fibrosis in India: a cross sectional study. Educ Adm Theory Pract. (2024) 30(5):4750–6. 10.53555/kuey.v30i5.3516

[26] Shen YW Shih YH Fuh LJ Shieh TM. Oral submucous fibrosis: a review on biomarkers, pathogenic mechanisms, and treatments. Int J Mol Sci. (2020) 21(19):7231. 10.3390/ijms21197231 33008091 PMC7582467

[27] Wollina U Verma SB Ali FM Patil K. Oral submucous fibrosis: an update. Clin Cosmet Investig Dermatol. (2015) 8:193–204. 10.2147/CCID.S80576 25914554 PMC4401336

[28] Chen X Xie H Guo J. Drug treatment for oral submucous fibrosis: an update. BMC Oral Health. (2023) 23(1):748. 10.1186/s12903-023-03488-9 37828490 PMC10568776

[29] Jency Evanjelin P Uma Maheswari TN Salam S Dhond SN Kulkarni V Sagili SR Clinical practice guidelines for management of different grades of oral submucous fibrosis. Community Pract. (2024) 21(05):561–570. 10.5281/zenodo.1118454

[30] Kamath VV. Surgical interventions in oral submucous fibrosis: a systematic analysis of the literature. J Maxillofac Oral Surg. (2015) 14(3):521–531. 10.1007/s12663-014-0639-3 26225039 PMC4510093

[31] Modak N Tamgadge S Tamgadge A Bhalerao S. Comparative study of clinical staging of oral submucous fibrosis with qualitative analysis of collagen fibers under polarized microscopy. Iran J Pathol. (2015) 10(2):111. 26351471 PMC4539763

[32] Khan S Chatra L Prashanth SK Veena KM Rao PK. Pathogenesis of oral submucous fibrosis. J Cancer Res Ther. (2012) 8(2):199–203. 10.4103/0973-1482.98970 22842361

[33] Ara SA Arora V Zakaullah S Raheel SA Rampure P Ashraf S. Correlation of habits and clinical findings with histopathological diagnosis in oral submucosal fibrosis patients. Asian Pac J Cancer Prev. (2013) 14(12):7075–7080. 10.7314/APJCP.2013.14.12.7075 24460253

[34] Gachinamath V Varma AS Gangavati R Huded P. Non-invasive laser technologies for early detection of oral cancer and dental diseases. Oral Sphere J Dent Health Sci. (2026) 2(2):120–124. 10.63150/osjdhs.2026.18

[35] Sawhney H Bhargava D Kashwani R Mishra R. Artificial intelligence as a tool for improving oral cancer outcomes. Arch Dent Res. (2023) 13(1):15–19. 10.18231/j.adr.2023.003

[36] Mehata FS Hammer JE. Text book of tobacco related oral mucous lesions and conditions India published by basic dental research unit. Bombay: Tata Institute of Fundamental Research (1993).

[37] Rajinikanth M Reddy VS Tirandas RK Chemalavagulapalli M Vidya KS. Proposed clinico-pathological classification of OSMF depending on review of different classification systems (1966–2015). Int J Res Applied Natl Soc Sci. (2016) 4:29–38.

